# Should We Reconstruct Coronal Plane Fractures in Multifragmentary Pertrochanteric Fractures? A Randomized Controlled Trial

**DOI:** 10.2106/JBJS.OA.26.00148

**Published:** 2026-07-02

**Authors:** Velmurugesan Purnaganapathi Sundaram, Nagashree Vasudeva, Agraharam Devendra, Yoga Bharathi, G.S. Balachandran, Sivakumar SP, Jayaramaraju Dheenadhayalan, Shanmuganathan Rajasekaran

**Affiliations:** 1 Department of Orthopaedics and Trauma, Ganga Medical Centre and Hospitals Pvt. Ltd, Coimbatore, India

## Abstract

**Introduction:**

Coronal plane fractures are increasingly identified in multifragmentary pertrochanteric (AO Foundation/Orthopaedic Trauma Association [AO/OTA] 31-A2) fractures, yet their clinical significance is under-researched. The aim of this study was to evaluate whether wire augmentation during Proximal Femur Nail Antirotation Asia (PFNA-II) fixation improves functional outcomes—regain of abductor strength and modified Harris Hip Score (mHHS)—compared with PFNA-II alone at 18-month follow-up. The secondary outcomes such as time to union, pain scores (Visual Analog Scale [VAS]), and complications were also assessed.

**Methodology:**

In this single-center, randomized controlled trial, 142 patients of Asian race and Indian ethnicity aged older than 60 years with AO-OTA 31-A2 fractures were treated using PFNAII, with or without wire augmentation for coronal fragments. Preoperative computed tomography (CT) assessed coronal fragment size. Among them, 116 patients who completed 18-month follow-up were analyzed.

**Results:**

Modified intention-to-treat analysis was performed. At 18 months, augmentation produced greater abductor strength (Δ 8.21%, 95% confidence interval, 4.4-12.83; p < 0.001) with no significant difference in mHHS (Δ 3.31 points, −1.3-7.9; p = 0.16). In fragments >50%, wire augmentation led to a clinically relevant 10% strength gain, exceeding the minimal clinically important difference (MCID) of 8.2%. However, VAS was similar between groups. Controls showed more varus collapse, likely due to a persisting posteromedial void when the fragment is not stabilized.

**Conclusion:**

Wire augmentation improved abductor strength recovery, especially when fragment size exceeded 50%, supporting its use in such cases. However, mHHS were similar between the groups.

**Level of Evidence:**

Level I Therapeutic. See Instructions for Authors for a complete description of levels of evidence.

## Introduction

AO Foundation/Orthopaedic Trauma Association (AO/OTA) 31-A2 fractures (A2.2 and A2.3) are pertrochanteric multifragmentary patterns characterized by lateral wall incompetency (≤20.5 mm^1^; Supplementary Fig. 1). They frequently include a posteromedial fragment with the lesser trochanter (LT), compromising medial support and increasing failure risk^2^. In addition, secondary coronal fracture line often originate from the summit of greater trochanter (GT) and extend along the trochanteric crest or toward the posteromedial cortex^3^. Cho et al. identified 2 distinct fracture patterns: 1 involving both the greater and lesser trochanters (GLT) and another involving both trochanters along with posteromedial cortex (greater and lesser along with posteromedial cortex [GLPC]), each occurring as a single fragment (corresponding to A2.2) or split type (A2.3; Supplementary Fig. 2)^3^. These fracture configurations are absent from Arbeitsgemeinschaft für Osteosynthesefragen (AO) classification but have gained recent attention^3-5^.

Large coronal fragments often compromise the nail entry point^6,7^. Furthermore, coronal lines traverse the midlateral cortex, compromising the cephalomedullary screw site. Nail insertion can displace posterior fragments, leaving the implant unsupported (Supplementary Fig. 3). When fracture lines exit more anteriorly, intraoperative lateral wall fracture may occur. This is often associated with anterior cortical disruption, resulting in an unsupported head-neck fragment^8^. This can lead to sagittal nail toggle and eventual loss of fracture reduction^9^. A biomechanical study has shown that posterolateral fractures interfering lag screw insertion increase instability. They suggested surgical strategies to minimize the coronal swing motion and changes in the neck-shaft angle^10^.

In coronal split patterns, the gluteus medius exerts lateral and superior pull, while short external rotators pull it posteriorly, creating tensile forces that risk displacement^11^. This leaves the fragment unreduced, and can result in malunion (Supplementary Fig. 4), shorten the abductor lever arm, and alter the length-tension relationship, producing lurch. Abductor weakness, already prevalent in the elderly, is worsened by trochanteric fracture^12^. This leads to longer rehabilitation, reliance on walking aids, reduced quality of life, increased fall risk, and long-term functional decline^13^. Despite their clinical significance and key concerns including open deformity of posterior fragment, sagittal instability, malunion, and abductor weakness, coronal plane fractures remain poorly addressed in current treatment protocols.

The aim of the study was to evaluate whether wire augmentation during Proximal Femur Nail Antirotation Asia (PFNA-II) fixation improves functional outcomes in 31-A2 fractures—regain of abductor strength and modified Harris Hip Score (mHHS; coprimary outcomes)—compared with PFNA-II alone at 18-month follow-up. The secondary objectives were to assess time to union, pain scores (Visual Analog Scale [VAS]), and complications and explore whether the benefit of wire augmentation varies with coronal fragment size. We hypothesized that anatomical reconstruction of coronal fragments using wire augmentation improves abductor strength recovery and mHHS, especially in patients with larger coronal fragments.

## Materials and Methods

### Study Design and Setting

This parallel, single center, randomized controlled trial was conducted at a Level 1 trauma center from January 2022 to December 2023. Ethical approval was obtained from the institutional review board, and the trial was registered with the Clinical Trial Registry of India (CTRI/2022/02/040473).

### Participants-Inclusion and Exclusion Criteria

**Fig. 1:**
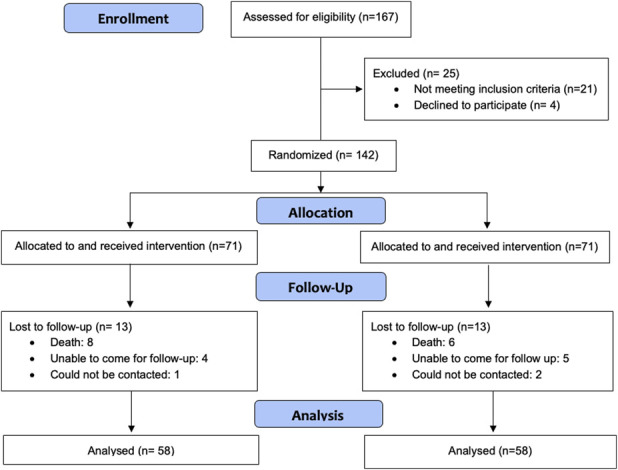
Flow diagram of patient enrollment, allocation, follow-up, and analysis (Consolidated Standards of Reporting Trials Guideline).

Patients aged older than 60 years with AO-OTA 31 A2 fractures were included. All participants belonged to Asian race and Indian ethnicity (self-reported). Exclusion criteria comprised AO 31A1 or 31A3 fractures, pathological fractures, previous proximal femur fractures, hip arthritis, multiple fractures, high surgical risk (American Society of Anesthesiologists ≥4), and impaired preinjury ambulation. A Consolidated Standards of Reporting Trials flow chart (Fig. 1) details patient selection. Written informed consent was obtained for both surgery and trial participation at the time of enrollment for the trial.

### Randomization and Blinding

Patients were randomized using a computer-generated randomization table (simple randomization) and assigned via serially numbered, sealed envelopes to ensure allocation concealment. One author handled enrollment and group assignment throughout the study. The intervention group received coronal fragment reconstruction using wiring and PFNA-II fixation, and the control group received PFNA-II alone. All procedures were performed by 2 senior orthopaedic surgeons (>10 years of experience) using a standardized technique to ensure consistency across all procedures. Surgeons were informed of group allocation just before surgery. Owing to the nature of the intervention, surgeon blinding was not feasible—a common limitation in surgical trials. However, this was an outcome assessor–blinded trial. Functional assessments were performed by a physiotherapist who was blinded to the randomization.

### Treatment Groups

Patients were placed supine on a fracture table. Traction was applied to assist in fracture reduction. Adhesive hip drapes with a blood collection bag connected to suction were used to measure intraoperative blood loss. Total blood loss was measured by adding the blood collected in the suction apparatus and the blood absorbed by surgical mops. Surgical duration was also recorded.

#### Intervention Group

A direct lateral approach was used to expose the GT. The posterior fragment was mobilized with a bone hook and the fracture was reduced using bone spikes, clamps, Steinmann pins, and K-wires. Reduction was considered acceptable if at least 2 of the following 4 criteria were met^14,15^

- Neck shaft angle (NSA): Restoration of NSA or 5-10° valgus
- Lateral view: less than 20° of angulation
- Medial and anterior cortical continuity
- Reduction variance: neutral or positive variance

A 3.5-mm drill was used to create an anteroposterior hole for a stainless-steel wire securing the anterior and posterior fragments. A second wire was passed around the shaft below the LT. In GLPC fractures, the posteromedial cortex was reduced and incorporated into the cerclage. Wires were intertwined in a “figure of 8” configuration and held loosely. Fixation was completed using a long PFNA-II with a helical blade, ensuring Tip-Apex Distance <25 mm^16,17^. Wires were then tightened, buried, and the wound closed over a drain. This configuration serves to stabilize the posterior coronal split of the GT by bringing the fragments together and resist varus deforming forces through a tension band mechanism during abduction.

#### Control Group

After positioning on the traction table, reduction was assessed under image intensification. If satisfactory, closed nailing was performed using standard steps; if inadequate, the fracture was opened and reduced as described above before proceeding with nailing

### Postoperative Protocol

Postoperatively, all patients had radiograph and computed tomography (CT) imaging. Bone mineral density scans identified osteoporosis, and patients were treated accordingly. Drains were removed after 48 hours or once output was <50 ml. Both groups followed the same rehabilitation protocol. Postoperatively, in-bed mobilization began on day 0. On day 1, assisted hip and knee range-of-motion exercises and transfers to standing or chair were initiated based on pain tolerance and clinical stability. Full weight-bearing with a walker started on day 2. Patients were discharged once the wound was dry and they could walk with a walker.

### Follow-Up

Patients were assessed in person and were contacted via telephone to remind them of the follow-up visits. Follow-up were performed at 6 weeks, 3 months, monthly until 6 months, and then every 6 months up to 18 months. Each visit included radiographs, evaluation of pain scores, and functional assessment at 6, 12, and 18 months (mHHS and abductor strength recovery).

### Data Collection

Baseline variables included age, sex, body mass index, and bone mineral density (*T* score). Preoperative radiographs identified fracture side and measured the neck-shaft angle of the opposite hip. CT scans were assessed for fracture classification using the Arbeitsgemeinschaft für Osteosynthesefragen system and coronal plane classification by Cho et al.^3^.

### Measurement of the Coronal Fragment Size

**Fig. 2:**
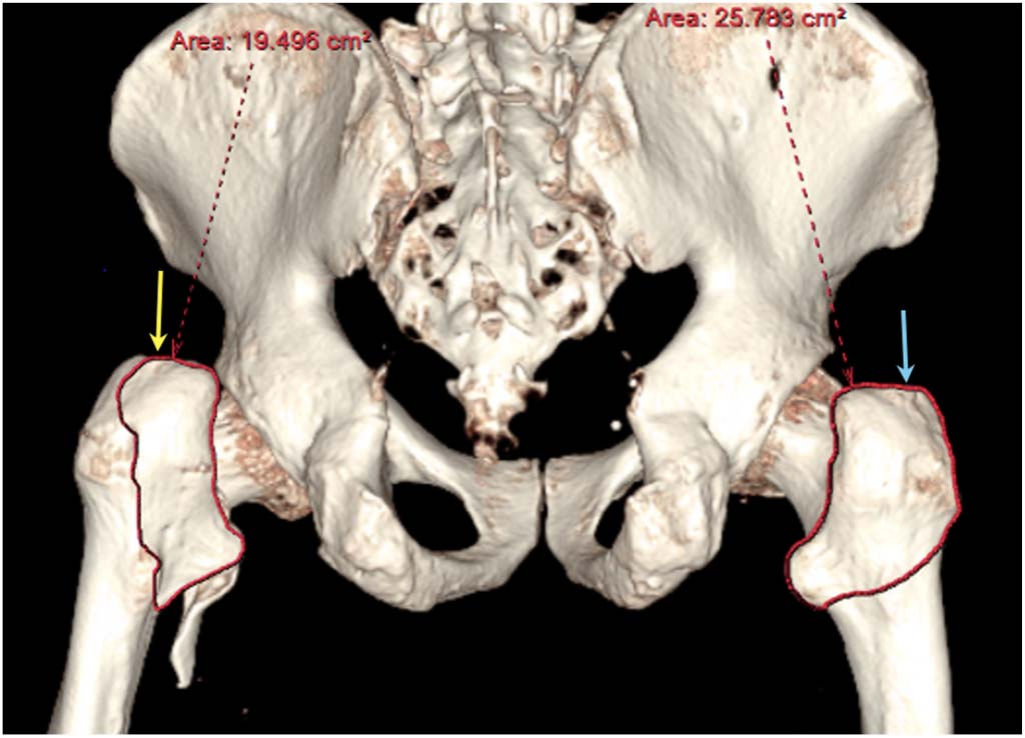
Measurement of the size of the fragment when compared with the normal side. For single large fragments (GLT or GLPC), the area was measured by outlining the fragment(Yellow arrow). On the contralateral normal side, the GLT were outlined on posterior 3D views, delineated by the intertrochanteric ridge, to define the trochanteric mass (Blue arrow). Fragment size is calculated as (fragment area/total trochanteric area) × 100. GLPC = greater and lesser along with posteromedial cortex, and GLT = greater and lesser trochanters.

Coronal fragment size was assessed on 3D-reconstructed CT images (Volume Rendering Technique, Picture Archiving and Communication System) using surface area analysis (Fig. 2). The posterior fragment was manually outlined with the polygon tool to obtain the area; split fragments were traced separately and summed. The trochanteric mass area on the contralateral side was measured, and fragment size was expressed as a percentage to minimize the individual bone size variation. For example, a fragment area of 19.49 cm^2^ and contralateral area of 25.78 cm^2^ yields fragment size to be 75.6%.

Postoperative radiographs and CT scans assessed reduction quality and intraoperative lateral wall breakage—defined as new fracture lines at the lateral cortex. NSA restoration was calculated as the absolute difference between NSA on operated side and uninjured side on radiograph. Postoperative transfusions were recorded. Pain scores were recorded on day 2 and at discharge.

### Outcome Measures

#### Primary Outcome Variables: 2 Coprimary Outcomes at 18 Months

**Fig. 3:**
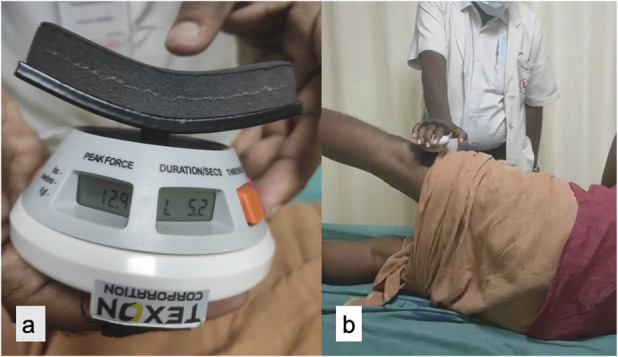
**Fig. 3-A** Hand-held dynamometer for the measurement of abductor strength. **Fig. 3-B** Patients were made to lie down in a lateral position and instructed to perform maximal voluntary isometric contractions in 3 trials, with 15-second rest intervals between attempts to minimize fatigue. The highest value was recorded. The same procedure was repeated on the opposite leg.

- Hip abductor strength: Measured using a handheld dynamometer in the side-lying position with the pad placed against the lateral femoral condyle (Fig. 3)^13,18,19^. Muscle strength recovery was expressed as a percentage of strength regained in the affected limb compared with the normal limb (referred to as abductor strength hereafter, unit: percentage points.
- mHHS: Assesses hip-related disability, including pain, function (gait and activities of daily living), deformity, and range of motion. The mHHS ranges from 0 to 100, with higher scores indicating better hip function. It is validated for pertrochanteric fracture outcomes treated with intramedullary nail fixation in the Indian population^20^.

### Secondary Outcome Variables

- Fracture union: assessed using the Radiographic Union Score for Hip (RUSH) on AP and lateral radiographs. A score ≥20 indicated union^21^. Time to union was recorded as the first follow-up visit at which this score was achieved. Clinically, we assessed painless weight-bearing and absence of local tenderness^22^.
- Pain: Measured using the VAS. Patients indicated their hip pain on that day on a 10-cm scale with numerical markings, where 0 represented no pain and 10 the worst imaginable pain, marking the point that best reflected their level.
- Functional outcomes including abductor strength and mHHS at 6 and 12 months.
- Complications: Varus collapse (>10° change in NSA), Wire breakage, blade backout, or implant failure.

### Protocol Modifications

- Abductor strength testing was finalized as the primary outcome after trial registration but before enrolling the first participant and was prospectively measured in all patients.
- The SF-12, initially planned as a secondary outcome, was excluded as many elderly patients had difficulty understanding and responding, so patient-reported outcome measures were not assessed.
- Follow-up was extended from 12 months to 18 months for a comprehensive assessment of healing, complications, and functional recovery.

### Statistical Analysis

#### Sample Size Estimation

Sample size was calculated using the Harris Hip Score (HHS) as the primary outcome, referencing Huang et al., who reported mean HHS of 96.4 ± 2.9 for PFN with cerclage and 93.1 ± 3.2 for PFN alone at 1-year follow-up^23^. Using this effect size, with 5% significance and 80% power, the WHO calculator estimated 128 patients (64 per group). Allowing for 10% attrition, the target sample size was 142 (71 per group). We chose a smaller effect for planning to avoid an underpowered trial if true effects were modest

### Data Analysis

Data were entered into Microsoft Excel and analyzed using SPSS v22 (IBM, Somers, NY, USA). Categorical variables were presented as frequencies/proportions and compared using the χ^2^ or Fisher exact tests and continuous variables as mean ± SD and compared using independent *t* tests. Wilcoxon signed rank test assessed intragroup changes over time.

All randomized participants were analyzed according to their assigned groups (modified intention-to-treat). Linear mixed-effects models were fitted for hip abductor strength and mHHS, with fixed effects for treatment group, time (6, 12, and 18 months), and treatment × time interaction, and a participant-level random intercept. Models were adjusted for age, sex, and AO/OTA fracture classification and estimated by maximum likelihood under a missing-at-random assumption. The adjusted treatment effect at 18 months was obtained using prespecified contrasts. To address higher attrition (18% vs. 10% anticipated), baseline and early postoperative parameters were compared between retained and lost patients. Group differences were assessed to evaluate the likelihood of attrition bias.

Multiplicity across the two 18-month coprimary tests was controlled using Holm-Bonferroni (family-wise α = 0.05); we report Holm-adjusted p-values and 95% confidence intervals. A per-protocol sensitivity repeated the adjusted models in complete cases; there were no crossovers or major deviations; the per-protocol population differed from the intention-to-treat population only by loss to follow-up. Study sensitivity was summarized using the minimum detectable difference (MDD) at 18 months, derived from the attained sample size and the observed pooled SD.

For hip abductor strength, clinical relevance was assessed using an anchor-based minimal clinically important difference (MCID). A distribution-based threshold (0.5 × SD) was calculated for supportive interpretation only. MCID was evaluated as a binary responder outcome at 18 months. Responder proportions were compared between groups using the χ^2^/Fisher tests with risk difference and 95% confidence interval (CI). An exploratory subgroup (coronal fragment ≥50% vs. <50%) assessed treatment group vs. fragment-size interaction and multiple linear regression were performed. A two-tailed p-value of <0.05 was considered statistically significant.

## Results

Between January 2022 and October 2022, 142 patients underwent surgery; 12 were lost to follow-up and 14 died, leaving 116 (58 per group) for final analysis (follow-up completed by May 2024). Baseline demographics (age, sex, fracture side/classification, *T*-scores) are enumerated in Table I.

**TABLE I:** Demographic Characteristics of the Participants

Parameter	Intervention group	Control group
Number	58	58
Age in yr, mean (SD)	71.07 ± 11.50	74.62 ± 11.46
Sex distribution, n (%)		
Men	25 (43.1)	20 (34.5)
Women	33 (56.9)	38 (65.5)
BMI, mean (SD)	23.15 ± 3.85	22.61 ± 3.64
Side, n (%)		
Left	22 (37.9)	18 (31)
Right	35 (60.3)	40 (69)
T-Score	−2.49 ± 0.69	−2.33 ± 0.88
Fracture characteristics, n (%)		
AO Classification		
2.2	38 (65.5)	26 (44.8)
2.3	20 (34.5)	32 (55.2)
Cho et al. classification		
GLT Fragment	33 (56.9)	23 (39.7)
GLPC Fragment	25 (43.1)	35 (60.3)

AO = Arbeitsgemeinschaft für Osteosynthesefragen, BMI = body mass index, GLPC = greater and lesser along with posteromedial cortex, and GLT = greater and lesser trochanters.

The intervention group had significantly longer surgical duration, greater blood loss, higher drain output, and more transfusions (Supplementary Table I). Intraoperative lateral wall break occurred in 19 patients (16.37%; 8 in intervention, 11 in controls; p = 0.13). Postoperative reduction quality was comparable between the groups (Supplementary Table I).

The primary outcome parameters of hip abductor strength and mHHS at 18 months, along with VAS pain scores, were analyzed using linear mixed-effects models (Table II). Models incorporated fixed effects for treatment group, time, and treatment × time interaction, with participant-level random intercepts. Adjusted estimates from linear mixed-effects models showed that at 18 months, augmentation produced greater hip abductor strength than control (marginal mean difference 8.21%, 95% CI, 4.4-12.83; Holm-adjusted p < 0.001). By contrast, the between-group difference in mHHS at 18 months was not significant (3.31 points, 95% CI, −1.3 to 7.9; Holm-adjusted p = 0.16).Within-group analysis showed a significant improvement in abductor strength from 6 months to 18 months in the augmentation group (p = 0.01), whereas no significant change was observed in the control group (p = 0.11).

**TABLE II:** Estimated Marginal Means (EMMs) for Primary and Secondary Outcomes (Mixed-Effects Models)

Parameter, mean ± SD	EMM Intervention group	EMM Control group	Δ (Intervention–Control)	95% CI	p
Primary outcome measures					
Abductor strength at 18 mo* (%)	85.96 ± 7.97	77.75 ± 12.52	+8.21	4.40 to 12.83	<0.001^†^
Modified Harris Hip Score at 18 mo*	81.50 ± 11.41	78.19 ± 13.76	+3.31	−1.30 to 7.90	0.16^†^
Secondary outcome measures					
Mean time to union in mo (RUSH ≥20)	4.81 ± 2.59	4.44 ± 1.32	+0.37	−0.22 to 0.96	0.22
Pain scores-VAS, (range)					
D 2	4.13 ± 1.84(2-6)	5.83 ± 2.14(2-7)	−1.70	−2.43 to −0.97	0.03
At discharge	1.59 ± 0.72 (0-4)	1.88 ± 0.88 (1-5)	+0.29	−0.16 to 0.74	0.05
6 mo	1.47 ± 1.04(0-5)	1.78 ± 1.61(0-5)	−0.31	−0.76 to 0.14	0.22
12 mo	1.12 ± 1.21(0-5)	1.36 ± 1.58(0-4)	−0.24	−0.69 to 0.21	0.29
18 mo	0.97 ± 1.30(0-3)	1.14 ± 1.68(0-4)	−0.17	−0.63 to 0.28	0.53
Abductor strength (%)					
6 mo	77.46 ± 12.76	70.62 ± 14.43	+6.84	1.29 to 14.39	0.02
12 mo	81.22 ± 10.91	73.72 ± 13.86	+7.50	3.25 to 11.76	<0.001
Modified Harris Hip Score					
6 mo	72.00 ± 13.23	67.71 ± 14.98	+4.29	−0.95 to 9.53	0.10
12 mo	77.17 ± 12.31	72.48 ± 13.94	+4.69	−0.09 to 9.47	0.06

Primary analysis: Modified intention-to-treat (all randomized patients with ≥1 postbaseline assessment).

Models: Linear mixed-effects models with random intercepts for participant; fixed effects for treatment, time (6, 12, 18 months), and treatment × time

Interpretation: Higher values indicate better outcome for abductor strength and mHHS; lower values indicate less pain for VAS.

EMM = Estimated Marginal Means, mHHS = modified Harris Hip Score, RUSH = Radiographic Union Score for Hip, VAS = Visual Analog Scale

* Coprimary outcomes (Holm-Bonferroni applied) at 18 months.

† Holm-adjusted p-values at 18 months.

Secondary outcomes: Time to radiological union did not differ significantly between the 2 groups (p = 0.22). Similarly, no significant between-group differences were observed for VAS pain at any time point or for time to union (all p > 0.05).

### Clinical Significance and MCID Analysis of Hip Abductor Strength

Clinical significance for hip abductor strength was assessed using an anchor-based MCID. Using mHHS ≥80 at 18 months to represent a good/excellent functional outcome as the anchor^20^, the corresponding anchor-based MCID for abductor strength was estimated to be approximately 8.2%. A distribution-based threshold (0.5 × SD of 18 months strength) was ≈5.5%, supporting clinical and statistical significance. Responders were defined as participants achieving a ≥8% improvement in hip abductor strength from 6 to 18 months. At 18 months, a greater proportion of patients in the augmentation group achieved the prespecified MCID compared with controls (71.7% vs. 56.2%), corresponding to an absolute risk difference of 15.5% (95% CI, −9.9% to 39.0%; χ^2^ p = 0.092).

Although the sample size allowed for 10% attrition, the actual rate was 18% due to higher mortality in trochanteric fractures^24^. Comparison of retained (n = 116) and excluded (n = 26) patients showed no significant differences in demographics, fracture type, intraoperative factors, or early radiographic parameters (Supplementary Table II), reducing the risk of attrition bias. Group comparisons remained valid and preserved the integrity of the primary outcome analysis.

As a measure of statistical sensitivity, the MDD for hip abductor strength at 18 months was 5.46%, based on the pooled SD of 10.50% and the attained sample size (n = 58 per group) and observed variability. The adjusted between-group difference exceeded this threshold, indicating adequate sensitivity to detect the observed effect. For mHHS, the pooled SD at 18 months was 12.64, yielding an MDD of 6.56; the observed between-group difference of 3.31 points did not exceed this threshold and was not statistically significant after Holm correction. This difference was well below published MCID values for mHHS (approximately 16-20 points) consistent with the neutral mHHS finding. Per-protocol sensitivity analyses reproduced the intention-to-treat inference (abductor strength significant; mHHS not significant).

### Subgroup Analysis of Abductor Strength Based on Fragment Size

Abductor strength was analyzed using a predefined cutoff of 50% fragment size. Augmentation produced greater abductor strength at 18 months in patients with ≥50% fragments (Δ 10.76 percentage points [pp]; 95% CI 5.67 to 15.85; p < 0.001), whereas there was no material difference for <50% fragments (Δ 3.24 pp; 95% CI, −1.67-8.15; p = 0.13; Supplementary Table III). By contrast, mHHS did not differ significantly between treatment groups in either subgroup (<50%: Δ 0.81 points; 95% CI, −8.01 to 9.63; p = 0.86; ≥50%: Δ 4.26 points; 95% CI, −1.18-9.71; p = 0.13).

Multiple linear regression assessed the association of fragment size, augmentation wiring, and their interaction with abductor strength recovery. The fragment size (%) was a significant predictor (p = 0.01), indicating poorer recovery with larger fragments. Wiring alone was not a significant predictor, but its interaction with fragment size was significant, suggesting that augmentation may mitigate the negative impact of larger fragment size on abductor strength recovery. By contrast, neither fragment size, cerclage, nor their interaction were significantly associated with mHHS, indicating no effect modification for functional outcomes (Supplementary Table IV and Supplementary Fig. 5).

### Complications

Three early surgical site infections occurred (2 in intervention group), all managed with debridement, wound wash, and intravenous antibiotics. One patient with augmentation had delayed union but remained asymptomatic, achieving full healing by 18 months (Supplementary Fig. 6). Wire breakage occurred in 2 cases, both asymptomatic postunion. GT fragmentation was seen in 1 patient in intervention group; 2 in control group had proximal trochanter displacement with abductor lurch, managed with physiotherapy. Varus collapse occurred in 7 cases in control group and 3 in intervention group.

## Discussion

The main finding of our study was the significant improvement in abductor when the coronal fractures were reconstructed. The effect was more pronounced when the size of the fragment was >50%. However, mHHS was not significantly different between the groups. The detectable-difference thresholds (5.46% for abductor strength and 6.56 points for mHHS) demonstrate that the observed improvement in abductor strength exceeded the study’s statistical sensitivity at 18 months, whereas the mHHS difference did not, consistent with the neutral mHHS findings.

The AO classification of trochanteric fractures reflects increasing complexity. Stable A1 fractures are easily treatable, whereas A2 and A3 are challenging. In A3 fractures with lateral wall break, augmentation with wires, screws, or plates is the standard of care^25-28^. However, managing A2 fractures poses dilemma as coronal plane lines often lead to loss of posterolateral and posteromedial support, making them highly unstable. Heydar et al. found that loss of posterolateral and posteromedial support independently predicted nail failure, with complications such as loss of femoral offset and abductor lever arm^29^. Despite this, reconstruction of trochanteric fragments lacks consensus and is left to the surgeon’s discretion.

Cerclage augmentation has shown benefits in unstable intertrochanteric fractures. Kulkarni et al. reported better functional outcomes and fewer complications in 154 patients treated with cerclage or lag screws; the nonaugmented group had 7.8% screw cutout and 6.4% back-out due to poor lateral wall support^26^. Babhulkar et al. found that cerclage augmentation improved alignment during reduction and nail insertion, leading to better outcomes and fewer fixation failures in unstable trochanteric fractures^30^. Gadegone et al. noted that while PFN addresses posteromedial defects, lateral support remains insufficient without augmentation, recommending cerclage or screws to restore lateral wall integrity and improve outcomes^31^.

We reconstructed the coronal fragments using the dynamic tension band principle, unlike cerclage, which provides static circumferential compression(Supplementary Fig. 7). The wire loop in the anteroposterior direction facilitates anatomical reduction, and the “figure-of-eight” configuration converts tensile forces into compression across the fracture site. During functional loading, the wire loop acts like a pulley, redirecting forces to maintain fragment apposition, neutralize lateral translation, and resist varus collapse. This dynamic mechanism likely explains improved abductor strength, particularly in cases with large coronal fragments. Stronger abductors enhance gait, reduce reliance on walking aids, and maintain pelvic stability. It may prevent hip arthritis, a consequence of chronic abductor insufficiency^32,33^.

This is the first study to evaluate fragment size as a determinant of outcomes in unstable intertrochanteric fractures. Larger fragments involving both posterolateral and posteromedial cortices increase instability by promoting sagittal nail toggle and loss of medial support^2^. Interaction analysis showed that wire augmentation mitigates the adverse effect of large fragment size where mechanical stability is compromised. Therefore, patients with fragments size >50% are more likely to benefit from wire augmentation.

Another notable finding was the increased incidence of varus collapse in the nonaugmented group due to inadequate support on the lateral wall. Fractures with posteromedial cortex involvement leave residual voids despite valgus reduction, predisposing to collapse during weight-bearing if not stabilized^34^ (Supplementary Fig. 8). This results in limb shortening, altered lever arm, and loss of femoral offset—key determinants of hip function. Therefore, optimizing initial stability is therefore essential to avoid future complications, especially critical in frail patients where additional procedures are challenging.

In our study, patients with augmentation reported reduced early postoperative hip pain, likely due to the stable construct reducing micromotion at the fracture site. By contrast, mobility of unstabilized fragments may provoke pain and hinder rehabilitation. As union progressed, fragment mobility decreased and pain subsided, resulting in similar final pain scores across groups. Though more invasive and time-consuming, wire augmentation improved mechanical stability, functional recovery, and reduced fixation failure risk. The main concern is potential vascular injury during wire placement; however, adherence to standardized protocols minimizes this risk, as supported by literature^35^. Therefore, the decision to use wire augmentation should be guided by a careful risk-benefit analysis tailored to each individual case.

### Limitations

This study has several limitations. The 18-month follow-up may not capture late complications such as hip arthritis, necessitating longer-term studies. Excluding high-risk patients (American Society of Anesthesiologists ≥4), multiple fractures, and preexisting hip pathology improved safety and reduced confounding but limits generalizability to broader patient populations. Surgeon blinding was not feasible due to procedural differences, possibly introducing bias; however, standardized protocols were followed, and outcomes were assessed by a blinded physiotherapist. Attrition was largely due to mortality. Participants retained vs lost were similar at baseline, supporting the MAR assumption for missing data, although some residual bias cannot be excluded. Protocol modifications occurred when the SF-12 questionnaire was omitted due to poor patient comprehension, restricting assessment of patient-reported outcomes. Also, the trial registry listed mHHS as primary outcome. However, abductor strength was designated as a coprimary outcome before enrolment.

## Conclusion

Augmentation of fixation with wiring significantly improved abductor muscle strength, particularly when coronal fragment size >50%. However, mHHS were similar between the groups. These results support the selective use of wire augmentation in appropriate patients, provided the potential benefits outweigh the associated risks.

## Ethics Approval

Approval was obtained from the Institutional ethics committee. The procedures used in this study adhere to the tenets of the Declaration of Helsinki.

## Consent to Participate and Publish

Informed consent was obtained from all individual participants included in the study.

## Funding

This project (Project no AOTAP21-41) was Supported by AO Trauma Asia Pacific under AO Foundation Research Grants 2021.

## Accessibility of the Trial Protocol

Clinical Trial Registry of India https://ctri.nic.in/Clinicaltrials/pubview.php.

## Accessibility of Data

The datasets generated and/or analyzed during the current study are available from the corresponding author on reasonable request.

## Appendix

Supporting material provided by the authors is posted with the online version of this article as a data supplement at jbjs.org (http://links.lww.com/JBJSOA/B236, http://links.lww.com/JBJSOA/B237, http://links.lww.com/JBJSOA/B238, http://links.lww.com/JBJSOA/B239, http://links.lww.com/JBJSOA/B240, http://links.lww.com/JBJSOA/B241, http://links.lww.com/JBJSOA/B242, http://links.lww.com/JBJSOA/B243, http://links.lww.com/JBJSOA/B244, http://links.lww.com/JBJSOA/B245, http://links.lww.com/JBJSOA/B246, http://links.lww.com/JBJSOA/B247).
